# Consciousness alterations in a cohort of young Swiss men: Associations with substance use and personality traits

**DOI:** 10.3389/fpsyt.2022.1056159

**Published:** 2023-01-04

**Authors:** Marianthi Lousiana Deligianni, Joseph Studer, Gerhard Gmel, Yasser Khazaal, Nicolas Bertholet

**Affiliations:** ^1^Addiction Medicine, Department of Psychiatry, Lausanne University Hospital (CHUV) and University of Lausanne, Lausanne, Switzerland; ^2^Service of Adult Psychiatry North-West, Department of Psychiatry, Lausanne University Hospital and University of Lausanne, Lausanne, Switzerland; ^3^Research Department, Addiction Switzerland, Lausanne, Switzerland; ^4^Centre for Addiction and Mental Health, Institute for Mental Health Policy Research, Toronto, ON, Canada; ^5^Faculty of Health and Social Science, University of the West of England, Bristol, United Kingdom; ^6^Research Centre, Montreal University Institute of Mental Health, Montreal, QC, Canada

**Keywords:** altered states of consciousness, psychedelics, substance use, young men, personality

## Abstract

**Background:**

Substance-induced consciousness alterations (CA) have mainly been studied among users of psychedelics but not among people using street drugs.

**Aims:**

Explore occurrences of three different types of substance-induced CA [ego dissolution (ED), visual pseudo-hallucinations (VPH), anxiety/paranoia (A/P)] and their perceived influences on life, together with their associations with substance use and personality correlates in a general population sample of 25-year-old men.

**Methods:**

2,796 young Swiss men lifetime substance users completed a self-report questionnaire including history of use (never, former, and current) of different substances categories (psychedelics, cocaine, psychostimulants, ecstasy, MDMA, and other drugs), substance-induced ego dissolution (ED), visual pseudo-hallucinations (VPH) and anxiety/paranoia (A/P), the influence of these CA experiences on life, and personality traits (sensation seeking, sociability, anxiety-neuroticism, and aggression–hostility).

**Results:**

32.2% reported at least one CA (i.e., ED, VPH or A/P), with 20.5% reporting ED, 16.7% VPH, and 14.6% A/P. Former and current use of psychedelics and ketamine was significantly associated with occurrences of all three types of CAs and with a positive influence of CA on life. Associations between the former and current use of other substances and the different types of CA were less consistent, and perceived influences on life were not statistically significant. Sociability was negatively associated with occurrences of all three types of CA. Positive associations were found between anxiety–neuroticism and ED and A/P, between aggression–hostility and A/P, and between sensation seeking and ED and VPH.

**Conclusion:**

This study supports the potential for psychedelics to induce CAs perceived as beneficial to life among people using street drugs, possibly reflecting the mechanism underlying the therapeutic potential of psychedelics.

## Introduction

Altered states of consciousness (ASC) refer to any conditions characterized by a significant temporary deviation from an individual's usual, subjective, conscious experience or psychological functioning (1). These deviations can include not only changes in mood or motor activity (as might be expected using alcohol or tranquilizers) but also unusual experiences regarding oneself and one's surroundings (1). Acute intoxication with psychotropic substances, such as psychostimulants (e.g., methamphetamine and cocaine), hallucinogens (e.g., psychedelics, cannabis, and MDMA), or alcohol and opioids, is known to transiently alter consciousness, undoubtedly contributing to their recreational or spiritual use (2). Substance-induced ASC can be described using three core dimensions: “oceanic boundlessness” (OBN) refers to an ecstatic state associated with positive mood and feelings of serenity and unity; “dread of ego dissolution” (DED) refers to unpleasant anxious states and paranoid ideation, commonly described by people using drugs as a “bad trip;” and “visionary restructuralization” (VRS), referring to altered perceptions, hallucinations, illusions, and synesthesia (3). Although psychotropic drugs share a common capacity to alter consciousness, they vary dramatically in tolerability. Individual responses can vary from meaningful pleasurable states to rather overwhelming stressful experiences (“bad trips”), or even to clinically significant psychotic or other psychiatric disorders among susceptible individuals (4–7). Growing evidence suggests that substance-induced psychosis may persist after acute substance use, withdrawal or abstinence, leading to a novel and entirely separate clinical entity of substance-related exogenous psychosis (8). A recent study by the European Drug Emergencies Network found that the frequency of psychotic episodes during acute drug intoxication varied considerably between drugs, with amphetamine being the drug most frequently implicated (9).

Contrary to amphetamine, classic psychedelics [i.e., serotonin 2A receptor agonists such as lysergic acid diethylamide (LSD), psilocybin, DMT, and mescaline] seem to present a relatively low potential for adverse effects on users and society compared to other substances (i.e., low physical and social harm potential and substance use disorder development) (10). For instance, a longitudinal study involving distinct groups of people in Switzerland using psychedelics, MDMA, psychostimulants, and cannabis found no differences in mental health between users of psychedelics and subjects who used no substances; however, people using other substances were associated with poorer mental health (11). Similarly, one large-scale population-based study found no significant associations between the use of psychedelics and any adverse mental health outcomes (12). There is also no significant association between the use of psychedelics and the risk of developing a substance use disorder (4), contrary to many other substances (10, 13). Notwithstanding, the recent gain in popularity of psychedelics (14) and the renewed interest in their potential therapeutic properties it seems important to define the conditions for their safe use, given the risk of challenging experiences. Nonetheless, it should be noted that in epidemiological studies, the use of psychedelics is often associated with other substance use (12). This should be considered when interpreting independent associations.

Substance-induced ASC have mostly been studied among people using psychedelics, following recent therapeutic interest in these drugs. Although some of these states could be linked to potential therapeutic uses for psychedelics (15, 16), they are not observed among all users. Contrary to psychedelics, other psychotropic substances, like cannabis or amphetamine, have mostly been examined from a public health perspective due to the risk of transitioning toward psychosis or a substance use disorder (5, 17, 18). Indeed, cannabis use has been linked to more dissociative and positive symptoms among first-episode psychosis patients (19), and more visual processing disturbances among patients presenting prodromal or acute psychotic symptomatology compared to healthy controls (18).

Psilocybin and ketamine have been found to present similar overlapping mechanisms of action and effects, ranging from positive mystical states to negative psychotic experiences, whereas MDMA has mostly been linked to positive affective states, with little anxiety, few thought disturbances, and no hallucinations resulting from common recreational doses among healthy volunteers (3). Recent research attempting to predict the factors influencing the nature of individuals' responses to psychotropic drugs underlined the contribution of contextual, sociodemographic, and personal differences (20–23). The dose of a substance likely affects the responses to it, as Earleywine et al. (24) found that high doses of cannabis could induce “oceanic boundlessness” comparable to psilocybin.

Personality traits have been postulated to be among the most crucial determinants of acute response to psychedelics, with trait neuroticism being associated with increased occurrences of adverse experiences among people using classic psychedelics (25, 26) or MDMA (23). A Swiss study among people receiving psilocybin in controlled experimental settings found that being hallucinogen naive, reporting moderate alcohol and cannabis consumption, and having experienced few psychological problems in the past few weeks were associated with pleasurable effects and fewer visual alterations, whereas young age and trait sociability were associated with more impaired control and cognition and more audio-visual synesthesiae respectively (27).

To the best of our knowledge, most recent studies exploring occurrences of ASC during acute substance use were conducted in selected samples, such as convenience internet surveys of naturalistic use or clinical pilot trials involving selected samples. Furthermore, most of them focused on one substance at a time, notably psychedelics (22–25, 27, 28).

Thus, little is known about substance-induced ASC and their correlates in the general population. A better understanding of substance-induced ASC may help us to better characterize factors predicting the nature of individual responses.

The present study is exploratory. Its primary aim was to describe occurrences of three different types of transient substance-induced consciousness alterations (CA), namely ego dissolution (ED), visual pseudo-hallucinations (VPH) and anxiety/paranoia (A/P), corresponding to specific aspects of the three core ASC dimensions of OBN, VRS and DED respectively, and their perceived influence on life among a non-clinical sample of 25-year-old men. Its secondary aim was to explore how history of use (i.e., never, former, and current use) of different categories of psychotropic drugs and personality characteristics were associated with occurrences of different types of substance-induced CA and the perceived influence on life those experiences had had on our subjects.

## Methods

### Enrollment procedure and participants

This study used data from the Cohort Study on Substance Use Risk Factors (C-SURF). Study participants were recruited between August 23, 2010, and November 15, 2011, at three of Switzerland's six army recruitment centers, covering 21 of its 26 cantons. Army recruitment is mandatory in Switzerland, and 98% of all 19-year-old Swiss males participate, allowing a large sample of the male population to be reached. Study procedures occurred independently of army involvement or eligibility to serve. Participants completed a baseline questionnaire at the age of about 20, followed by two follow-up assessments at about 21 and 25. C-SURF research was approved by Lausanne University Medical School's Clinical Research Ethics Committee (Protocol No. 15/07). A total of 7,556 young men gave their written consent to participate in the study at those army recruitment centers. Of these, 5,987, 6,020, and 5,516 completed the baseline, first follow-up, and second follow-up questionnaires, respectively. The present study's sample consists of the participants who completed all three assessments and who reported having used substances in at least one of the following categories during their life: (a) cannabis and spice; (b) psychedelics (natural, e.g., psilocybin, peyote, mescaline, salvia divinorum, or synthetic, e.g., LSD, PCP/angel dust, 2-CB and 2-CI, ketamine, and DXM); (c) cocaine and other psychostimulants (speed, amphetamine, methamphetamine, and crystal meth); (d) ecstasy and MDMA; and (e) other drugs (i.e., poppers, inhalants, GHB, heroin, and bath salts). Of the 4,981 who completed the baseline and the two follow-up assessments, 4,845 (97.3%) had no missing values on substance-use measures, and statistical analyses were carried out using the data from the 2,847 (58.8%) men who reported having used at least one substance in one of the above five categories during their life. After listwise deletion, the final analytical sample comprised 2,796 cases (98% of the eligible sample).

### Measures

#### History of substance use

The baseline questionnaire asked participants whether they had used any substance in the five above-mentioned categories in their lifetime—categories formed based on pharmacological agents, effects, and modes of action. The first and second follow-up questionnaires asked whether they had used these substances in the previous 12 months.

We also assessed tobacco and alcohol use. For alcohol, the frequency of heavy episodic drinking [defined as drinking six or more standard drinks on a single occasion; (29)] was measured. Based on measures from the baseline, first, and second follow-up assessments, a variable reflecting the history of substance use at the age of about 25 (second follow-up) was created for each category of substance users: never users (no use reported at any assessment), former users (use at baseline or the first follow-up, but no use at the second follow-up), and current users (users at the second follow-up, but also possibly at baseline and the first follow-up).

#### Substance-induced consciousness alterations

Participants lifetime experiences of CA under the influence of drugs were assessed at about 25 years old using items adapted from the Abnormal Mental States (APZ) questionnaire (1), a widely used self-reporting scale for assessing subjective past experiences of ASC (30). To limit the length of the very extensive C-SURF questionnaire and participants' response burden, we adapted the single most representative item in the APZ questionnaire corresponding to specific aspects for each core dimension—OBN, DED, and VRS, namely ego dissolution (ED), anxiety/paranoia (A/P) and visual pseudo-hallucinations (VPH) respectively. Participants were asked to answer “Yes” or “No” to the following statements: “In your life, under the influence of a drug, have you ever experienced the following?”

^*^ The boundaries between yourself and your surroundings seemed to blur (ED aspect of OBN).
^*^ You felt threatened or afraid, without being able to say exactly why (A/P aspect of DED).
^*^ You saw things that you know were not real (VPH aspect of VRS).

#### Perceived influence on life of experiences of substance-induced CA

Participants reporting at least one substance-induced CA were asked to evaluate the impact that experience had had on their life by answering the following question: “In your opinion, what influence has this type of experience had on your life in general?” Possible answers were: “Very positive” (scored +2), “Somewhat positive” (+1), “Neither positive nor negative” (0), “Almost negative” (−1), and “Very negative” (−2).

#### Personality traits

At about 25 years old, the cross-cultural version of the Zuckerman–Kuhlman Personality Questionnaire (31) was used to assess the three personality traits of aggression–hostility, sociability, and neuroticism–anxiety. Participants answered ten true-or-false statements for each trait. Mean scores were computed if respondents answered at least eight items for each trait. Means were then scaled up (multiplying by 10) to the original metric (sums). Sensation seeking was measured using the Brief Sensation-Seeking Scale (32), with eight items rated on a five-point Likert scale [from “Strongly disagree” (scored +1) to “Strongly agree” (+5)]. Mean scores were computed if at least six items were answered. Means were then scaled up (multiplying by 8) to the original metric (sums).

#### Sociodemographic variables

Sociodemographic variables measured at about 25 years old were used to adjust the analyses. These included age, linguistic region (German or French), highest educational level achieved (primary, ≤ 9 years of schooling; vocational 9–12 years; and post-secondary ≥13 years, including high school, which can be 12 years in some cantons).

### Statistical analyses

Descriptive statistics were used to describe the sample characteristics and differences in experiences of CAs as a function of subjects' history of substance use, personality, and sociodemographic characteristics. Logistic regression models were used to test the associations between history of substance use and personality characteristics, and CAs. Differences between types of CA in the influence of their experience on life were tested using independent-samples' *t*-tests for participants reporting only a single CA. One-sample *t*-tests were used to test whether the influence of each CA experience on life (in participants reporting only a single CA) was significantly different from zero. Finally, linear regression models were used to test the associations between history of substance use and personality characteristics, and the influence of their experiences of CAs on their life. These logistic and linear regression calculations tested both bivariate and fully adjusted models (i.e., history of substance use, personality characteristics, and sociodemographic variables). All analyses were performed using SPSS version 27 (33). The significance level was set to alpha = 0.05.

## Results

### Sample characteristics

Tables 1, 2 show the characteristics of the sample of respondents reporting the use of at least one substance from the five above-mentioned categories during their lifetime. At 94.8%, the majority reported having used cannabis or spice. For each of the four other categories of psychedelics and ketamine, cocaine and other psychostimulants, ecstasy and MDMA, and other drugs, the prevalence of lifetime use was about 22%. A total of 900 (32.2% of the sample) participants reported at least one CA (i.e., VPH, ED or A/P). The mean (SD) number of CA reported was 0.52 (0.86; not tabulated). ED was the most frequently reported CA (20.5%), followed by VPH (16.7% of the sample) and A/P (14.6% of the sample). Overall, the perceived influence of experiences of CA on participants' lives was slightly positive [0.27 (0.99)] and statistically different from zero [*t*(899) = 8.17, *p* < 0.001].

**Table 1:** Prevalence of CAs, history of substance use, education, and linguistic region.

	**Total**	**VPH**	**ED**	**A/P**
**CA**, ***N*** **(%)**				
Yes	900 (32.2)	466 (16.7)	574 (20.5)	407 (14.6)
No	1896 (67.8)	2330 (83.3)	2222 (79.5)	2389 (85.4)
**History of cannabis and spice use**, ***N*** **(%)**				
Never	146 (5.2)	11 (7.5)	20 (13.7)	8 (5.5)
Former	1229 (44.0)	138 (11.2)	178 (14.5)	166 (13.5)
Current	1421 (50.8)	317 (22.3)	376 (26.5)	233 (16.4)
**History of ecstasy and MDMA use**, ***N*** **(%)**				
Never	2203 (78.8)	231 (10.5)	316 (14.3)	252 (11.4)
Former	235 (8.4)	86 (36.6)	93 (39.6)	68 (28.9)
Current	358 (12.8)	149 (41.6)	165 (46.1)	87 (24.3)
**History of cocaine and other psychostimulant use**, ***N*** **(%)**				
Never	2175 (77.8)	233 (10.7)	314 (14.4)	255 (11.7)
Former	268 (9.6)	77 (28.7)	95 (35.4)	61 (22.8)
Current	353 (12.6)	156 (44.2)	165 (46.7)	91 (25.8)
**History of psychedelics and ketamine use**, ***N*** **(%)**				
Never	2161 (77.3)	197 (9.1)	304 (14.1)	234 (10.8)
Former	396 (14.2)	125 (31.6)	146 (36.9)	107 (27.0)
Current	239 (8.5)	144 (60.3)	124 (51.9)	66 (27.6)
**History of other drugs use (poppers, inhalants, GHB, heroin, etc.)**, ***N*** **(%)**				
Never	2134 (76.3)	282 (13.2)	364 (17.1)	259 (12.1)
Former	461 (16.5)	106 (23.0)	135 (29.3)	99 (21.5)
Current	201 (7.2)	78 (38.8)	75 (37.3)	49 (24.4)
**History of heavy episodic alcohol drinking**, ***N*** **(%)**				
Never	699 (25.0)	83 (11.9)	117 (16.7)	83 (11.9)
Former	727 (26.0)	117 (16.1)	143 (19.7)	107 (14.7)
Current	1370 (49.0)	266 (19.4)	314 (22.9)	217 (15.8)
**History of tobacco use**, ***N*** **(%)**				
Never	374 (13.4)	38 (10.2)	51 (13.6)	25 (6.7)
Former	795 (28.4)	94 (11.8)	122 (15.3)	87 (10.9)
Current	1627 (58.2)	334 (20.5)	401 (24.6)	295 (18.1)
**Education**, ***N*** **(%)**				
Primary	85 (3.0)	23 (27.1)	26 (30.6)	21 (24.7)
Vocational	1021 (36.5)	185 (18.1)	199 (19.5)	140 (13.7)
Post-secondary	1690 (60.4)	258 (15.3)	349 (20.7)	246 (14.6)
**Linguistic region**, ***N*** **(%)**				
French	1579 (56.5)	259 (16.4)	319 (20.2)	274 (17.4)
German	1217 (43.5)	207 (17.0)	255 (21.0)	133 (10.9)

CA, consciousness alterations; VPH, visual pseudo-hallucinations; ED, ego dissolution; A/P, anxiety/paranoia.

**Table 2:** Means of age, personality traits, and the influences on life of experiences with CA for the total sample and as a function of CA.

	**Total**	**VPH**		**A/P**		**ED**	

		**No**	**Yes**	**No**	**Yes**	**No**	**Yes**
Age, M (SD)	25.45 (1.25)	25.44 (1.26)	25.43 (1.14)	25.41 (1.23)	25.64 (1.30)	25.43 (1.23)	25.49 (1.28)
Sensation seeking, M (SD)	3.20 (0.76)	3.13 (0.74)	3.48 (0.73)	3.17 (0.75)	3.31 (0.76)	3.13 (0.74)	3.44 (0.75)
Aggression–hostility, M (SD)	3.89 (2.17)	3.83 (2.15)	4.11 (2.20)	3.77 (2.12)	4.50 (2.29)	3.80 (2.14)	4.18 (2.22)
Sociability, M (SD)	5.01 (2.21)	5.05 (2.20)	4.79 (2.25)	5.09 (2.18)	4.56 (2.33)	5.10 (2.16)	4.65 (2.34)
Anxiety–neuroticism, M (SD)	2.33 (2.22)	2.27 (2.17)	2.60 (2.41)	2.12 (2.09)	3.48 (2.55)	2.18 (2.14)	2.85 (2.43)
Influence of experiences with CA on life, M (SD)	0.27^a^ (0.99)	–	0.48^b^ (0.84)	–	−0.16^b^ (0.97)	–	0.37^b^ (0.98)

CA, consciousness alterations; VPH, visual pseudo-hallucinations; A/P, anxiety/paranoia; ED, ego dissolution; M, mean; SD, standard deviation; ^*a*^Only participants reporting at least one CA included (n = 900); ^*b*^Only participants reporting one CA included (VPH, n = 153; A/P, n = 126; ED, n = 206).

### Associations between CA, history of substance use, and personality traits

**Figure 1:**
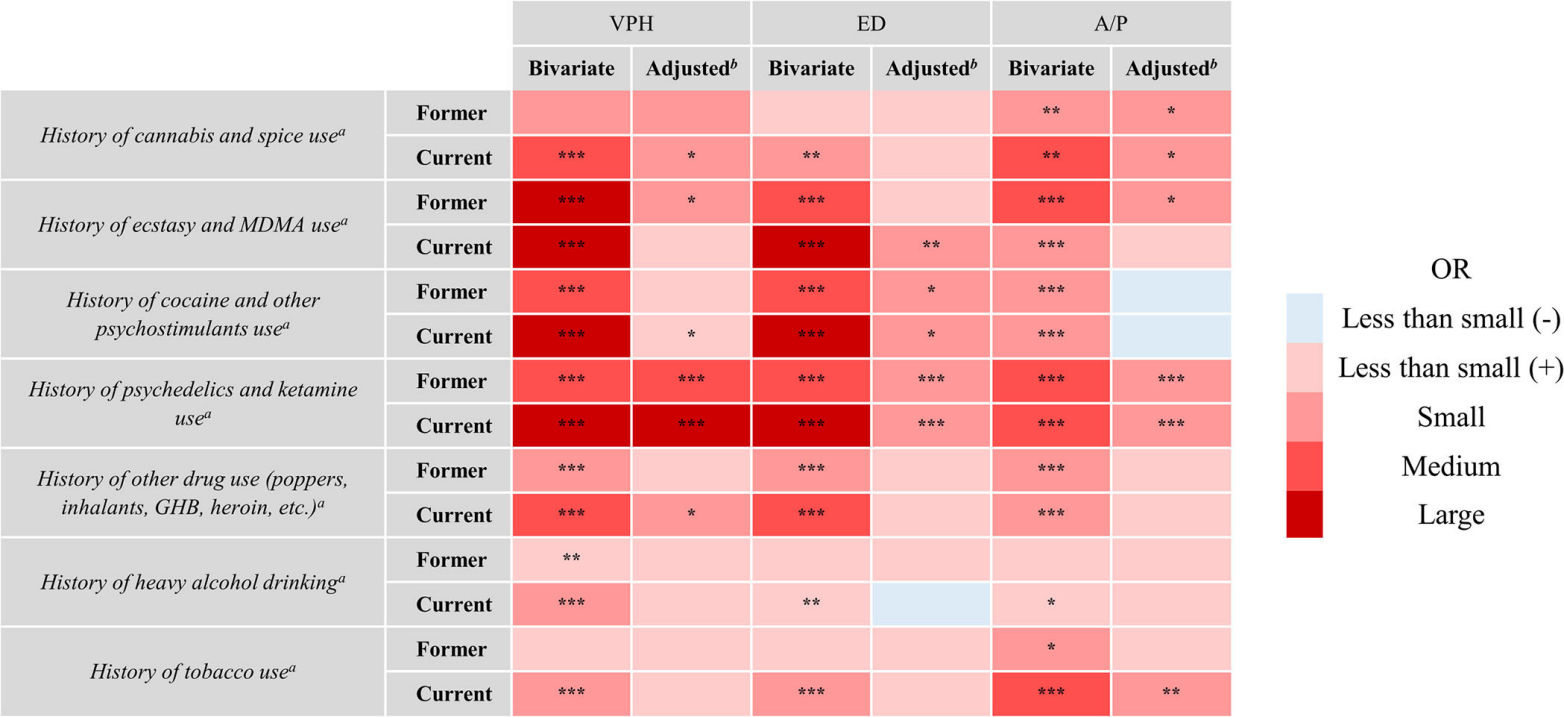
Size and the statistical significance of the odds ratios of logistic regression models investigating the associations between a history of substance use and CA. CA, consciousness alterations; VPH, visual pseudo-hallucinations; ED, ego dissolution; A/P, anxiety/paranoia; ^***a***^Reference: never; ^b^Adjusted for history of substance use, personality, age, education, and linguistic region. *p < 0.05, **p < 0.01, ***p < 0.001. Size of odds ratio according to Chen et al. (34) when rate of outcome is ~5% in the reference group; Less than small (–): 0.65 < OR < 1.00; Less than small (+): 1.00 < OR < 1.52; Small: 1.52 < OR < 2.74; Medium: 2.74 < OR < 4.72; Large: OR > 4.72.

Table 1 reports the prevalence of never, former, and current substance use as a function of CA. Means of personality traits and age as a function of CA are reported in Table 2, and the results of the logistic regression models investigating associations between CA, history of substance use, and personality traits are reported in Figure 1 (see Supplementary material 1 for exact ORs and 95% confidence intervals) and Table 3 respectively.

**Table 3:** Results of logistic regression models investigating the associations between personality traits and CA.

	**VPH**		**ED**		**A/P**	

	**Bivariate**	**Adjusted** *^*a*^*	**Bivariate**	**Adjusted** *^*a*^*	**Bivariate**	**Adjusted** *^*a*^*
	**OR (95% CI)**	**OR (95% CI)**	**OR (95% CI)**	**OR (95% CI)**	**OR (95% CI)**	**OR (95% CI)**
**Personality**				
Sensation Seeking	**1.93 (1.67–2.24)**	**1.34 (1.14–1.59)**	**1.79 (1.57–2.05)**	**1.38 (1.19–1.61)**	**1.29 (1.11–1.48)**	1.12 (0.95–1.32)
Aggression–Hostility	**1.06 (1.01–1.11)**	0.99 (0.94–1.05)	**1.08 (1.04–1.13)**	1.02 (0.97–1.07)	**1.16 (1.11–1.22)**	**1.08 (1.02–1.14)**
Sociability	**0.95 (0.91–0.99)**	**0.93 (0.88–0.98)**	**0.91 (0.88–0.95)**	**0.91 (0.87–0.95)**	**0.90 (0.86–0.94)**	**0.94 (0.89–0.99)**
Anxiety–Neuroticism	**1.07 (1.02–1.11)**	1.02 (0.97–1.08)	**1.13 (1.09–1.18)**	**1.09 (1.04–1.14)**	**1.27 (1.22–1.32)**	**1.21 (1.16–1.27)**

CA, consciousness alterations; VPH, visual pseudo-hallucinations; A/P, anxiety/paranoia; ED, ego dissolution; ^a^Adjusted for history of substance use, personality, age, education, and linguistic region; p <0.05 in bold.

### Associations with visual pseudo-hallucinations

In our bivariate analyses, former and current use of drugs in each substance category were significantly associated with increased odds of VPH, except for the former use of cannabis and spice, and tobacco. For example, the odds of VPH were about 4.6 times greater among former users (odds ratio [OR] = 4.60; 95% confidence interval [CI], 3.55–5.95) and 15.1 times greater among current users (OR = 15.11, 95% CI, 11.22–20.35) of psychedelics and ketamine than among non-users. All personality traits were significantly associated with VPH: positively for sensation seeking, aggression–hostility, and anxiety–neuroticism, and negatively for sociability.

Regarding history of substance use, only associations with the former and current use of psychedelics and ketamine, former use of ecstasy and MDMA, current use of cannabis and spice, cocaine and other psychostimulants, and other drugs remained significant in the fully adjusted analyses, with ORs being more prominent for psychedelics and ketamine.

Regarding VPH's associations with personality traits, only sensation seeking and sociability remained significant after adjustment.

### Associations with ego dissolution

In our bivariate analyses, former and current use of drugs in each substance category were significantly positively associated with ED, except for the former use of cannabis and spice, tobacco, and heavy episodic alcohol drinking. All personality traits were significantly associated with ED: positively for sensation seeking, aggression–hostility, and anxiety–neuroticism, and negatively for sociability.

Regarding history of substance use, only associations with the former and current use of psychedelics and ketamine, and cocaine and psychostimulants, as well as current use of ecstasy and MDMA, remained significant in the fully adjusted analyses, with a greater OR for psychedelics and ketamine.

Adjustment had a minor effect on ED's associations with personality traits, except for the association with aggression–hostility, which became insignificant.

### Associations with anxiety/paranoia

In our bivariate analyses, former and current use of drugs in each category of substances were significantly associated with increased odds of A/P, except for former heavy episodic alcohol drinking. All the personality traits were significantly associated with A/P: positively for sensation seeking, aggression–hostility, and anxiety–neuroticism, and negatively for sociability.

Regarding history of substance use, only current tobacco use, former ecstasy and MDMA use, and former and current cannabis and spice, and psychedelics and ketamine use remained significant in our fully adjusted analyses, with ORs being greatest for cannabis and spice.

Except for sensation seeking, all the personality traits remained significantly associated with A/P after adjustment.

### The influence on life of experiencing CA

As reported in Table 2, the participants reporting only one CA (*n* = 485) perceived its influence on their life to have been positive for VPH [*n* = 153; *M* = 0.48; SD = 0.84; difference from zero *t*(152) = 7.09, *p* < 0.001] and ED [*n* = 206; *M* = 0.37; SD = 0.98; difference from zero *t*(205) = 5.46, *p* < 0.001]. For participants reporting A/P, influence on their life was perceived as slightly negative (*n* = 126; *M* = −0.16; SD = 0.97) but their scores were not significantly different from zero [*t*(125) = 1.83, *p* = 0.070]. These scores differed significantly between VPH and A/P [*t*(277) = 5.90, *p* < 0.001] and between ED and A/P [*t*(330) = 4.80, *p* < 0.001], but not between VPH and ED [*t*(357) = 1.11, *p* = 0.267].

Table 4 reports the results of our linear regression models investigating the associations between history of substance use, personality, and the influence on life that experiencing a CA had had. In bivariate analyses, former and current use of ecstasy and MDMA, psychedelics and ketamine, and current use of cocaine and other psychostimulants, and other drugs were associated with more positive influence on life, whereas former use of tobacco was negatively associated. Sensation seeking was significantly positively associated with experiencing a CA as having a positive influence on life (i.e., more positive), whereas the anxiety–neuroticism and aggression–hostility traits were significantly negatively (i.e., less positive) associated.

**Table 4:** Results from linear regression models investigating the associations between a history of substance use and the perceived influence of experiences of CA on life.

	**Bivariate**	**Adjusted^b^**
	**B (95% CI)**	**B (95% CI)**
**History of cannabis and spice use** ^a^		
Former	−0.27 (−0.14, 0.60)	−0.14 (−0.52, 0.23)
Current	0.21 (−0.17, 0.59)	0.18 (−0.19, 0.56)
**History of ecstasy and MDMA use** ^a^		
Former	**0.25 (0.07, 0.44)**	0.16 (−0.06, 0.38)
Current	**0.40 (0.25, 0.55)**	0.20 (−0.01, 0.40)
**History of cocaine and other psychostimulants use** ^a^		
Former	0.00 (−0.19, 0.20)	−0.19 (−0.40, 0.03)
Current	**0.33 (0.18, 0.48)**	−0.07 (−0.27, 0.14)
**History of psychedelics and ketamine use** ^a^		
Former	**0.18 (0.02, 0.34)**	**0.19 (0.02, 0.36)**
Current	**0.59 (0.43, 0.75)**	**0.36 (0.17, 0.55)**
**History of other drug use (poppers, inhalants, GHB**,		
**heroin, etc.)** ^a^		
Former	−0.01 (−0.17, 0.16)	−0.06 (−0.22, 0.11)
Current	**0.25 (0.05, 0.45)**	0.07 (−0.14, 0.28)
**History of heavy alcohol drinking** ^a^		
Former	0.10 (−0.10, 0.29)	0.00 (−0.18, 0.19)
Current	0.15 (−0.02, 0.32)	0.00 (−0.17, 0.17)
**History of tobacco use** ^a^		
Former	–**0.26 (–0.52, –0.01)**	−0.18 (−0.42, 0.07)
Current	−0.13 (−0.36, 0.10)	−0.19 (−0.42, −0.03)
**Personality**		
Sensation seeking	**0.21 (0.13, 0.30)**	0.09 (−0.00, 0.17)
Aggression–hostility	–**0.03 (–0.06, –0.01)**	−0.01 (−0.04, 0.02)
Sociability	−0.01 (−0.04, 0.02)	–**0.04 (–0.07, –0.01)**
Anxiety–neuroticism	–**0.07 (–0.10, –0.04)**	–**0.07 (–0.09, –0.04)**

^a^Reference: Never; ^b^Adjusted for history of substance use, personality, age, education, and linguistic region; p < 0.05 in bold.

However, in the fully adjusted model, the only associations that remained significant were the positive associations of former and current use of psychedelics and ketamine and the negative associations of anxiety–neuroticism. In addition, although not significant in bivariate analysis, the sociability trait's adjusted association was significantly negative.

## Discussion

The present study explored the occurrence of specific consciousness alterations (CA), namely ego dissolution (ED), visual pseudo-hallucinations (VPH) and anxiety/paranoia (A/P), corresponding to the three core ASC dimensions of OBN, VRS and DED respectively, their perceived influence on life, and how they correlated with personality traits in a cohort of young Swiss males prospectively followed from 20 to 25 years old.

To the best of our knowledge, this was one of the first general population studies on the topic.

About one third of our sample of people reporting the use of at least one drug during their lifetime reported at least one of the three CA in question. Participants felt that the experience of a substance-induced CA had had a positive influence on their life on average. However, when CA were assessed by type of experience, this was only true for ED and VPH. For those reporting A/P, however, the experience had had a slightly negative influence, but was not significantly different from zero.

Former and current use of psychedelics and ketamine was consistently associated with all three types of CA. Although their use was also associated with challenging experiences (i.e., A/P), compared with the other categories of substances, the use of psychedelics and ketamine showed their largest effect sizes for ED and VPH. Furthermore, in the fully adjusted model, the only category of substance use associated with CA having a significant positive influence on life was psychedelics including ketamine. These results may reflect a possible positive, long-lasting effect of psychedelics on wellbeing, as suggested by previous studies implementing interventions for healthy individuals using psychedelics (35). Studies among clinical samples have also shown that the quality of the experience was predictive of positive mental health outcomes among individuals presenting depressive and addictive disorders (15, 16, 36, 37). History of ecstasy and MDMA use was also associated with all three types of CA, but to a lesser extent than psychedelics use. Only former use of ecstasy and MDMA was associated with VPH and A/P, whereas only current use was associated with ED. Taken together, these findings are in line with those of Vollenweider (3), showing that both psychedelics, ketamine, and MDMA induced more ASC of each type than a placebo and that their effects were greater for psychedelics and ketamine. Contrary to existing literature suggesting high rates of acute toxicity and adverse mental health outcomes, notably substance-induced psychosis, among users of cocaine and other psychostimulants (9, 38–40), our sample revealed no significant associations between using these substances and A/P in the adjusted model. Instead, there were significant associations with CA generally experienced as positive (i.e., ED and VPH), which is consistent with their widespread recreational use. The association between former and current cannabis and spice use and A/P showed the largest effect size. Since cannabis is known to induce anxiety and panic (41), this association may reflect challenging, “bad trip” type experiences among users. Current tobacco use was also found to be an important predictor of A/P. To the best of our knowledge, no previous studies investigating ASC have found significant associations with tobacco use, although the use of at least one illicit drug has been associated with tobacco use (42). Nevertheless, regular tobacco use has been associated with an increased risk of psychotic-like experiences in the general population (43). This finding may reflect regular tobacco users' shared vulnerability to psychotic-like and A/P experiences under the influence of drugs, and this could be due to common underlying factors such as specific tobacco-use related effects or may result from an addiction-driven vulnerability (44, 45).

Consistent with previous studies showing associations between the traits of anxiety–neuroticism and aggression–hostility and challenging experiences under the influence of drugs (22, 23, 25, 46), we found associations between these personality traits and A/P. One possible explanation is that people with these personality traits are more prone to negative emotional reactions and stress sensitivity when faced with novel experiences, notably anxiety for those with the anxiety–neuroticism trait and fear and feelings of insecurity for those with the aggression–hostility trait. They are thus prone to experience substance-induced CA more negatively (47).

Sensation seeking was significantly positively associated with ED and VPH, probably reflecting users' expectations about the drugs used and a readiness to surrender to their potential psychotropic effects. Expectations and beliefs are known to influence experiences of ASC (46, 48).

In our sample, sociability was negatively associated with occurrences of VPH, ED, and A/P. This could be due to extroverts being more likely to focus on external experiences rather than internal ones (49) and thus being less perceptive to the psychotropic effects of drugs. This is compatible with clinical observations that people with schizoid traits, low sociability and high introversion may be more prone to experiment and search psychedelics and other novel psychoactive substances. Our study's associations between sociability and VPH, ED, and A/P were not consistent with a previous Swiss study among healthy volunteers receiving psilocybin in an experimental context, reporting no or not significant associations (27). To the best of our knowledge, associations between sociability and A/P and ED have never been reported previously. Differences might therefore have arisen due to different study contexts and designs and because our study was not focused exclusively on psilocybin use.

Interestingly, anxiety–neuroticism—a trait found to be associated with challenging experiences (25, 27), which explains its use as an exclusion criterion in some studies (27)—was also positively correlated with ED, rated positively in our sample. One possible explanation for this is that drug effects are not only influenced by drug and personality factors but also by pre-use mood and drug-session context, known as “set and setting” (50, 51). Nevertheless, a recent study on the effects of MDMA found that a positive mindset before MDMA use could explain its potential beneficial effects among people predisposed to anxiety (23). As mentioned above, however, trait anxiety–neuroticism was less likely to predict positive feelings about the experience of a CA.

To the best of our knowledge, this was the first study to explore the comparative occurrences of CA induced by different psychotropic drugs, correlations with personality traits, and the CA's perceived influence on substance users' lives in a large, non-selective sample. Contrary to previous ones, this study did not focus solely on specific substances, thus limiting the participation bias linked to individuals' substance preferences.

This study had some limitations. The study was exploratory, thus results should be confirmed by future studies. The sample consisted solely of young men, so the results cannot be generalized to women and other age groups. Furthermore, because all the data were self-reported, without biochemical or dose verification, substance use may have been under-reported because of social desirability bias or fear of stigma, despite anonymity (participants were explicitly and repeatedly informed that their answers were confidential). There is also the possibility of a wrong correspondence between what a person have thought to get and what a person actually gets (especially for controlled substances). In addition, the dose of a psychotropic substance is known to impact the nature of the experience, which seems to also depend on other individual (i.e., pre-drug experiences, expectations, and individual susceptibility to the effects of a given substance) and contextual factors (i.e., context of use, use with other substances). Consequently, various factors may have contributed to inferior rates of substance-induced CA compared to experimental studies, where these factors can be evaluated and manipulated. Different substances were analyzed by categories according to their action profile or prevalence (e.g., the “other drugs” group consisted of opioids and other substances with minimal prevalence in our sample); thus, there was some heterogeneity within the tested groups. Finally, CA were not evaluated using a validated measure. Although questions were formulated based on existing literature on ASC, our cohort study was not designed to specifically answer the present study's research question and, thus, due to the time constraints associated with the regular assessments, only a limited number of questions concerning specific aspects of ASC dimensions could be included. More precisely, we used items 13 (OBN), 80 (VRS), and 64 and 71 (DED) from the APZ questionnaire. Because our questions concerned specific limited aspects of ASCs, other specific aspects of ASCs (i.e., auditory, emotional, and cognitive) could not be evaluated, and thus the questions asked may not have been broad enough to cover the differences in psychoactive effects induced by different substance categories.

## Conclusion

To the best of our knowledge, the present study was the first to explore substance-induced consciousness alterations (CA) resulting from different commonly used drugs among a large, representative, population-based sample. It also explored correlations with users' personality traits and the positive or negative influences on their life that they attributed to their experience of a substance-induced CA.

Among the substance categories tested, psychedelics and ketamine, showed the largest effect sizes for two of the three aspects of ASC dimensions, namely ED for OBN and VPH for VRS, both of which were rated positively in our sample. Although associations were also significant for A/P, psychedelics, including ketamine, were the only substances significantly associated with the perception that experiencing a CA had had a positive influence on the user's life. These findings are compatible with the hypothesized therapeutic effects of psychedelics and ketamine, as suggested by previous experimental studies. However, the risk of challenging experiences not being negligible, there is further need to explore the determinants of the nature of individual responses and define the conditions for their safe and beneficial use.

## Data availability statement

The datasets presented in this study can be found in online repositories. The names of the repository/repositories and accession number(s) can be found below: https://zenodo.org/record/5469953#.Yr7wILdBwuU.

## Ethics statement

The studies involving human participants were reviewed and approved by Lausanne University Medical School's Clinical Research Ethics Committee (Protocol No. 15/07). The patients/participants provided their written informed consent to participate in this study.

## Author contributions

Conceptualization, design of the work, and data interpretation: MD, JS, GG, YK, and NB. Critical revising: JS, GG, YK, and NB. Analysis: MD and JS. Drafting: MD. Data curation, funding acquisition, and project administration: JS and GG. All authors contributed to this article and approved the submitted version.

## References

[1] Dittrich A. The standardized psychometric assessment of altered states of consciousness (Ascs) in humans. Pharmacopsychiatry. (1998) 31(S 2):80–4. 10.1055/s-2007-979351 9754838

[2] Johnson MW Hendricks PS Barrett FS Griffiths RR. Classic psychedelics: an integrative review of epidemiology, therapeutics, mystical experience, and brain network function. Pharmacol Therap. (2019) 197:83–102. 10.1016/j.pharmthera.2018.11.010 30521880

[3] Vollenweider FX. Brain mechanisms of hallucinogens and entactogens. Dialog Clin Neurosci. (2001) 3:265. 10.31887/DCNS.2001.3.4/fxvollenweider PMC3181663 22033605

[4] Johnson MW Richards WA Griffiths RR. Human hallucinogen research: guidelines for safety. J Psychopharmacol. (2008) 22:603–20. 10.1177/0269881108093587 18593734 PMC3056407

[5] Murrie B Lappin J Large M Sara G. Transition of substance-induced, brief, and atypical psychoses to schizophrenia: a systematic review and meta-analysis. Schizoph Bull. (2020) 46:505–16. 10.1093/schbul/sbz102 31618428 PMC7147575

[6] Starzer MSK Nordentoft M Hjorthøj C. Rates and predictors of conversion to schizophrenia or bipolar disorder following substance-induced psychosis. Am J Psychiatry. (2018) 175:343–50. 10.1176/appi.ajp.2017.17020223 29179576

[7] Martinotti G Merino Del Villar C Garcia Cordoba A Andrés Tubau L Castro Sánchez I Di Carlo F. Club drugs and psychiatric sequelae: an issue of vulnerability and previous psychiatric history. Int J Environ Res Public Health. (2021) 18:6944. 10.3390/ijerph18136944 34209645 PMC8297170

[8] Martinotti G De Risio L Vannini C Schifano F Pettorruso M Di Giannantonio M. Substance-related exogenous psychosis: a postmodern syndrome. CNS Spect. (2021) 26:84–91. 10.1017/S1092852920001479 32580808

[9] Vallersnes OM Dines AM Wood DM Yates C Heyerdahl F Hovda KE. Psychosis associated with acute recreational drug toxicity: a European case series. BMC Psychiatry. (2016) 16:293. 10.1186/s12888-016-1002-7 27538886 PMC4990880

[10] Van Amsterdam J Opperhuizen A Koeter M Van Den Brink W. Ranking the harm of alcohol, tobacco and illicit drugs for the individual and the population. Eur Addict Res. (2010) 16:202–7. 10.1159/000317249 20606445

[11] Rougemont-Bücking A Jungaberle H Scheidegger M Merlo MCG Grazioli VS Daeppen JB. Comparing mental health across distinct groups of users of psychedelics, Mdma, psychostimulants, and cannabis. J Psychoact Drugs. (2019) 51:236–46. 10.1080/02791072.2019.1571258 30836844

[12] Krebs TS Johansen PØ. Psychedelics and mental health: a population study. PLoS ONE. (2013) 8:e63972. 10.1371/journal.pone.0063972 23976938 PMC3747247

[13] Nutt D King LA Saulsbury W Blakemore C. Development of a rational scale to assess the harm of drugs of potential misuse. Lancet. (2007) 369:1047–53. 10.1016/S0140-6736(07)60464-4 17382831

[14] National Institute on Drug Abuse. Marijuana and Hallucinogen Use Among Young Adults Reached All Time-High in 2021. New York, NY: National Institute on Drug Abuse (2022). Available online at: https://nida.nih.gov/news-events/news-releases/2022/08/marijuana-and-hallucinogen-use-among-young-adults-reached-all-time-high-in-2021 (accessed November 23, 2022).

[15] Majić T Schmidt TT Gallinat J. Peak experiences and the afterglow phenomenon: when and how do therapeutic effects of hallucinogens depend on psychedelic experiences? J Psychopharmacol. (2015) 29:241–53. 10.1177/0269881114568040 25670401

[16] Roseman L Nutt DJ Carhart-Harris RL. Quality of acute psychedelic experience predicts therapeutic efficacy of psilocybin for treatment-resistant depression. Front Pharmacol. (2018) 8:974. 10.3389/fphar.2017.00974 29387009 PMC5776504

[17] Cooper ZD Haney M. Investigation of sex-dependent effects of cannabis in daily cannabis smokers. Drug Alcohol Depend. (2014) 136:85–91. 10.1016/j.drugalcdep.2013.12.013 24440051 PMC4518446

[18] Koethe D Gerth CW Neatby MA Haensel A Thies M Schneider U. Disturbances of visual information processing in early states of psychosis and experimental delta-9-tetrahydrocannabinol altered states of consciousness. Schizoph Res. (2006) 88:142–50. 10.1016/j.schres.2006.07.023 17005373

[19] Ricci V Ceci F Di Carlo F Lalli A Ciavoni L Mosca A. Cannabis use disorder and dissociation: a report from a prospective first-episode psychosis study. Drug Alcohol Depend. (2021) 229:109118. 10.1016/j.drugalcdep.2021.109118 34688166

[20] Atakan Z Bhattacharyya S Allen P Martín-Santos R Crippa JA Borgwardt SJ. Cannabis affects people differently: inter-subject variation in the psychotogenic effects of δ9-tetrahydrocannabinol: a functional magnetic resonance imaging study with healthy volunteers. Psychol Med. (2013) 43:1255–67. 10.1017/S0033291712001924 23020923

[21] Carhart-Harris RL Roseman L Haijen E Erritzoe D Watts R Branchi I. Psychedelics and the essential importance of context. J Psychopharmacol. (2018) 32:725–31. 10.1177/0269881118754710 29446697

[22] Haijen ECHM Kaelen M Roseman L Timmermann C Kettner H Russ S. Predicting responses to psychedelics: a prospective study. Front Pharmacol. (2018) 9:897. 10.3389/fphar.2018.00897 30450045 PMC6225734

[23] Studerus E Vizeli P Harder S Ley L Liechti ME. Prediction of Mdma response in healthy humans: a pooled analysis of placebo-controlled studies. J Psychopharmacol. (2021) 35:556–65. 10.1177/0269881121998322 33781103 PMC8155734

[24] Earleywine M Ueno LF Mian MN Altman BR. Cannabis-induced oceanic boundlessness. J Psychopharmacol. (2021) 35:841–7. 10.1177/0269881121997099 33779383

[25] Barrett FS Johnson MW Griffiths RR. Neuroticism is associated with challenging experiences with psilocybin mushrooms. Personal Individ Differ. (2017) 117:155–60. 10.1016/j.paid.2017.06.004 28781400 PMC5540159

[26] Bouso JC Dos Santos RG Alcázar-Córcoles MÁ Hallak JEC. Serotonergic psychedelics and personality: a systematic review of contemporary research. Neurosci Biobehav Rev. (2018) 87:118–32. 10.1016/j.neubiorev.2018.02.004 29452127

[27] Studerus E Gamma A Kometer M Vollenweider FX. Prediction of psilocybin response in healthy volunteers. PLoS ONE. (2012) 7:e30800. 10.1371/journal.pone.0030800 22363492 PMC3281871

[28] Carbonaro TM Bradstreet MP Barrett FS MacLean KA Jesse R Johnson MW. Survey study of challenging experiences after ingesting psilocybin mushrooms: acute and enduring positive and negative consequences. J Psychopharmacol. (2016) 30:1268–78. 10.1177/0269881116662634 27578767 PMC5551678

[29] National Institute on Alcohol Abuse Alcoholism (NIAAA). Drinking Levels Defined. Bethesda, MD: NIAAA (2021). Available online at: https://www.niaaa.nih.gov/alcohol-health/overview-alcohol-consumption/moderate-binge-drinking (accessed Decemeber 12, 2021).

[30] Studerus E Gamma A Vollenweider FX. Psychometric evaluation of the altered states of consciousness rating scale (Oav). PLoS ONE. (2010) 5:e12412. 10.1371/journal.pone.0012412 20824211 PMC2930851

[31] Aluja A Rossier J García LF Angleitner A Kuhlman M Zuckerman M. Cross-cultural shortened form of the Zkpq (Zkpq-50-Cc) adapted to English, French, German, and Spanish languages. Person Individ Differ. (2006) 41:619–28. 10.1016/j.paid.2006.03.001

[32] Hoyle RH Stephenson MT Palmgreen P Lorch EP Donohew RL. Reliability and validity of a brief measure of sensation seeking. Person Individ Differ. (2002) 32:401–14. 10.1016/S0191-8869(01)00032-0

[33] IBM Corp. Ibm Spss Statistics for Windows, Version 27. Armonk, NY: IBM Corp. (2020).

[34] Chen H Cohen P Chen S. How big is a big odds ratio? Interpreting the magnitudes of odds ratios in epidemiological studies. Commun Stat Simul Comput. (2010) 39:860–4. 10.1080/03610911003650383

[35] Aday JS Mitzkovitz CM Bloesch EK Davoli CC Davis AK. Long-term effects of psychedelic drugs: a systematic review. Neurosci Biobehav Rev. (2020) 113:179–89. 10.1016/j.neubiorev.2020.03.017 32194129

[36] Dakwar E Anerella C Hart CL Levin FR Mathew SJ Nunes EV. Therapeutic infusions of ketamine: do the psychoactive effects matter? Drug Alcohol Depend. (2014) 136:153–7. 10.1016/j.drugalcdep.2013.12.019 24480515 PMC4091719

[37] Garcia-Romeu A Griffiths RR Johnson MW. Psilocybin-occasioned mystical experiences in the treatment of tobacco addiction. Curr Drug Abuse Rev. (2015) 7:157. 10.2174/1874473708666150107121331 25563443 PMC4342293

[38] Chang X Sun Y Zhang Y Muhai J Lu L Shi J. review of risk factors for methamphetamine-related psychiatric symptoms. Front Psychiatry. (2018) 9:603. 10.3389/fpsyt.2018.00603 30519197 PMC6251327

[39] Miró Ò Waring WS Dargan PI Wood DM Dines AM Yates C. Variation of drugs involved in acute drug toxicity presentations based on age and sex: an epidemiological approach based on european emergency departments. Clin Toxicol. (2021) 59:896–904. 10.1080/15563650.2021.1884693 33724118

[40] Sabe M Zhao N Kaiser S. A systematic review and meta-analysis of the prevalence of cocaine-induced psychosis in cocaine users. Prog Neuro-Psychopharmacol Biol Psychiatry. (2021) 109:110263. 10.1016/j.pnpbp.2021.110263 33524454

[41] Crippa JA Zuardi AW Martín-Santos R Bhattacharyya S Atakan Z McGuire P. Cannabis and anxiety: a critical review of the evidence. Hum Psychopharmacol. (2009) 24:515–23. 10.1002/hup.1048 19693792

[42] Conway KP Green VR Kasza KA Silveira ML Borek N Kimmel HL. Co-occurrence of tobacco product use, substance use, and mental health problems among adults: findings from wave 1 (2013–2014) of the population assessment of tobacco and health (path) study. Drug Alcohol Depend. (2017) 177:104–11. 10.1016/j.drugalcdep.2017.03.032 28582698 PMC5534376

[43] Saha S Scott JG Varghese D Degenhardt L Slade T McGrath JJ. The association between delusional-like experiences, and tobacco, alcohol or cannabis use: a nationwide population-based survey. BMC Psychiatry. (2011) 11:202. 10.1186/1471-244X-11-202 22204498 PMC3313864

[44] Zullino DF Manghi R Rathelot T Khan R Khazaal Y. Cannabis causes schizophrenia? So does nicotine. Addict Res Theory. (2010) 18:601–5. 10.3109/16066359.2010.489999

[45] Zullino DF Rathelot T Khazaal Y. Cannabis and psychosis. Lancet. (2007) 370:1540. 10.1016/S0140-6736(07)61653-5 17980730

[46] Aday JS Davis AK Mitzkovitz CM Bloesch EK Davoli CC. Predicting reactions to psychedelic drugs: a systematic review of states and traits related to acute drug effects. ACS Pharmacol Transl Sci. (2021) 4:424–35. 10.1021/acsptsci.1c00014 33860172 PMC8033773

[47] Jacobs N Van Os J Derom C Thiery E Delespaul P Wichers M. Neuroticism explained? From a non-informative vulnerability marker to informative person: context interactions in the realm of daily life. Br J Clin Psychol. (2011) 50:19–32. 10.1348/014466510X491397 21332518

[48] Polito V Langdon R Brown J. The experience of altered states of consciousness in shamanic ritual: the role of pre-existing beliefs and affective factors. Consc Cognit. (2010) 19:918–25. 10.1016/j.concog.2010.05.013 20558090

[49] Rossier J Aluja A Blanch A Barry O Hansenne M Carvalho AF. Cross–Cultural generalizability of the alternative five–factor model using the Zuckerman–Kuhlman–Aluja personality questionnaire. Eur J Person. (2016) 30:139–57. 10.1002/per.2045

[50] Carhart-Harris RL Nutt DJ. Serotonin and brain function: a tale of two receptors. J Psychopharmacol. (2017) 31:1091–120. 10.1177/0269881117725915 28858536 PMC5606297

[51] Hartogsohn I. Set and setting, psychedelics and the placebo response: an extra-pharmacological perspective on psychopharmacology. J Psychopharmacol. (2016) 30:1259–67. 10.1177/0269881116677852 27852960

