# Supplementary material for: Consciousness alterations in a cohort of young Swiss men: Associations with substance use and personality traits
Source: Front Psychiatry. 2023 Jan 4;13:1056159. doi: 10.3389/fpsyt.2022.1056159 (PMC9846235; doi:10.3389/fpsyt.2022.1056159)
Supplement: Supplementary file 1 [file Table_1.DOCX]

Supplementary Table 1. Results of logistic regression models investigating the associations between a history of substance use and CA

|  | VPH | | ED | | A/P | |
| --- | --- | --- | --- | --- | --- | --- |
|  | **Bivariate** | **Adjusted*^b^*** | **Bivariate** | **Adjusted*^b^*** | **Bivariate** | **Adjusted*^b^*** |
|  | **OR (95% CI)** | **OR (95% CI)** | **OR (95% CI)** | **OR (95% CI)** | **OR (95% CI)** | **OR (95% CI)** |
| *History of cannabis and spice use^a^* |  |  |  |  |  |  |
| Former | 1.55 (0.82–2.94) | 1.84 (0.92–3.68) | 1.07 (0.65–1.76) | 1.08 (0.63–1.86) | **2.69 (1.30–5.60)** | **2.63 (1.22–5.69)** |
| Current | **3.52 (1.88–6.60)** | **2.23 (1.12–4.44)** | **2.27 (1.39–3.69)** | 1.45 (0.85–2.48) | **3.38 (1.64–7.00)** | **2.31 (1.07–4.99)** |
| *History of ecstasy and MDMA use^a^* |  |  |  |  |  |  |
| Former | **4.93 (3.66–6.64)** | **1.73 (1.14–2.64)** | **3.91 (2.93–5.21)** | 1.43 (0.97–2.11) | **3.15 (2.31–4.30)** | **1.67 (1.07–2.59)** |
| Current | **6.09 (4.74–7.82)** | 1.43 (0.96–2.12) | **5.11 (4.02–6.49)** | **1.80 (1.27–2.58)** | **2.49 (1.89–3.27)** | 1.19 (0.78–1.81) |
| *History of cocaine and other psychostimulants use^a^* |  |  |  |  |  |  |
| Former | **3.36 (2.50–4.52)** | 1.26 (0.84–1.89) | **3.26 (2.47–4.30)** | **1.60 (1.11–2.31)** | **2.22 (1.62–3.04)** | 0.94 (0.61–1.45) |
| Current | **6.60 (5.14–8.48)** | **1.49 (1.00–2.21)** | **5.20 (4.09–6.62)** | **1.56 (1.09–2.24)** | **2.62 (1.99–3.43)** | 0.98 (0.64–1.50) |
| *History of psychedelics and ketamine use^a^* |  |  |  |  |  |  |
| Former | **4.60 (3.55–5.95)** | **2.88 (2.11–3.95)** | **3.57 (2.81–4.52)** | **2.14 (1.60–2.87)** | **3.05 (2.35–3.95)** | **2.11 (1.52–2.93)** |
| Current | **15.11 (11.22–20.35)** | **6.85 (4.78–9.81)** | **6.59 (4.97–8.73)** | **2.60 (1.83–3.68)** | **3.14 (2.29–4.30)** | **2.11 (1.41–3.15)** |
| *History of other drug use (poppers, inhalants, GHB, heroin, etc.)^a^* |  |  |  |  |  |  |
| Former | **1.96 (1.53–2.52)** | 1.01 (0.74–1.38) | **2.01 (1.60–2.54)** | 1.19 (0.90–1.58) | **1.98 (1.53–2.56)** | 1.34 (0.99–1.83) |
| Current | **4.17 (3.05–5.68)** | **1.62 (1.08–2.43)** | **2.89 (2.13–3.94)** | 1.20 (0.82–1.75) | **2.33 (1.65–3.30)** | 1.5 (0.99–2.28) |
| *History of heavy alcohol drinking^a^* |  |  |  |  |  |  |
| Former | **1.42 (1.05–1.93)** | 1.16 (0.83–1.62) | 1.22 (0.93–1.60) | 1.03 (0.77–1.38) | 1.28 (0.94–1.74) | 1.21 (0.87–1.68) |
| Current | **1.79 (1.37–2.33)** | 1.13 (0.82–1.54) | **1.48 (1.17–1.87)** | 0.98 (0.75–1.29) | **1.40 (1.06–1.83)** | 1.17 (0.86–1.59) |
| *History of tobacco use^a^* |  |  |  |  |  |  |
| Former | 1.19 (0.80–1.77) | 1.03 (0.67–1.59) | 1.15 (0.81–1.63) | 1.04 (0.72–1.52) | **1.72 (1.08–2.73)** | 1.29 (0.80–2.10) |
| Current | **2.28 (1.60–3.26)** | 1.19 (0.80–1.78) | **2.07 (1.51–2.84)** | 1.23 (0.87–1.75) | **3.09 (2.02–4.73)** | **1.90 (1.21–2.99)** |

Note. CA: Consciousness Alterations; VPH: Visual Pseudo-Hallucinations; A/P: Anxiety/Paranoia; ED: Ego Dissolution; ***^a^*** Reference: Never; ^b^ Adjusted for history of substance use, personality, age, education, and linguistic region; *p* < 0.05 in bold.
